# Ballistic Reconstruction of a Migrating Bullet in the Parapharyngeal Space

**DOI:** 10.1155/2015/245360

**Published:** 2015-12-07

**Authors:** David Bächinger, Stephan Bolliger, Gerhard F. Huber, Roman D. Laske

**Affiliations:** ^1^Department of Otorhinolaryngology, Head and Neck Surgery, University Hospital Zurich and University of Zurich, 8091 Zurich, Switzerland; ^2^Institute of Forensic Medicine, University of Zurich, 8057 Zurich, Switzerland

## Abstract

A 21-year-old male suffering from severe throat pain after being hit by a bullet in Syria claimed that he was shot through his eye and that the bullet subsequently descended behind his throat. Even though the first medical report stated that this course is implausible, meticulous workup provided evidence that the bullet might have entered the parapharyngeal space in a more cranial position than the one it was found eight months later. Our case highlights that bullets are able to move within the body, rendering ballistic reconstruction difficult. However, after removal of the bullet the patient's symptoms completely resolved.

## 1. Introduction

Injuries and cosmetic deformities following facial gunshot injuries (FGIs) are often serious, although rarely fatal [1]. In a recent case series, the most commonly injured structure in FGIs was the eye, with 31% (26/84) of patients suffering either unilateral or bilateral injury, and the most common residual problems included blindness and cranial nerve palsies [2]. Regardless of the entrance site, gunshot injuries to the head and neck additionally pose a particular risk to various vital structures encompassed in the parapharyngeal space resulting in direct injury and infection [3].

Upon presentation, treatment of gunshot injuries to the head and neck includes ATLS protocols and treating other life-threatening injuries. Contrast enhanced CT detects common and relevant injuries to the neck, especially fractures and vascular injuries [4]. Subsequently, wounds are assessed, foreign bodies are removed, and skeletal injuries are reduced [5]. However, morbidity associated with the removal of a foreign body, a bullet in particular, has to be factored in. Although there is some controversy on removing retained bullets, especially in cases with intra-abdominal injuries [6], there are certain situations where it is clearly indicated. If bullets are found in joints, in CSF, in the eye, and in proximity of a nerve or vessel, are visible or palpable at clinical examination, or would cause lead poisoning, removal is recommended [7]. Although rare, a possible complication is a spontaneous passive movement of the bullet causing neurovascular complications, previously described along the spinal cord, within blood vessels, the genitourinary tract, the gastrointestinal tract, and the lung [8–10].

## 2. Case Presentation

A 21-year-old Syrian male seeking asylum in Switzerland was referred to our tertiary centre with a history of a constantly sore throat causing sleep deprivation. Furthermore, he complained about increased pain on swallowing food and fluids. He reported that he had been wounded by a bullet eight months before in Syria, where he had been part of a Kurdish armed force. According to the patient, the bullet had glanced off a wall and had entered his right upper pharynx after perforating his left eye. After being hit, the patient suffered from anterograde amnesia covering one day. However, he was not provided appropriate medical treatment and the bullet was not removed. On the third day after the incident, the patient had been able to eat again despite serious pain and intermittent vomiting. Subsequently, the general condition improved but the patient suffered from a permanent foreign body sensation with pain behind the pharynx. Over several months he noticed a gradual, caudal migration of the symptoms.

**Figure 1:**
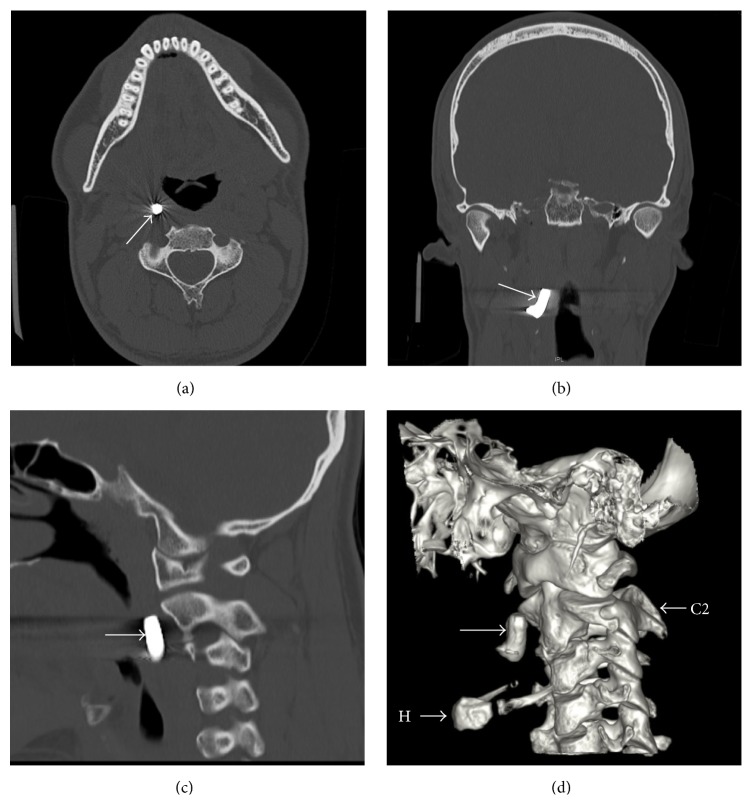
Axial (a), coronal (b), and sagittal (c) CT scans of the head and neck region showing the projectile in the parapharyngeal space, just ventrally to the longus colli muscle and medially to the carotid sheath. (d) A three-dimensional reconstruction (left lateral view). Arrow: projectile, H: hyoid, and C2: vertebra C2.

**Figure 2:**
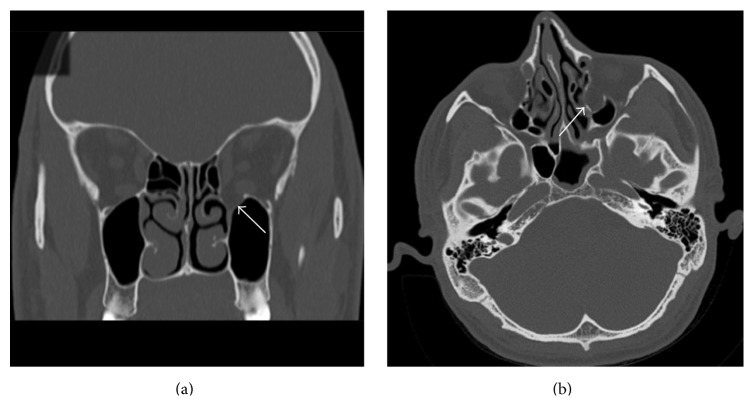
Coronal (a) and axial (b) CT scans showing the old dislocated fracture of the left medial orbital floor and wall.

Upon clinical examination, a foreign body was palpable through the mouth, just lateral to the right tonsil in the parapharyngeal space. The patient was found to have phthisis bulbi (i.e., a shrunken eye ball that lost its function) on the left side. Routine laboratory testing revealed a microcytic hypochromic anaemia and an iron and vitamin B12 deficiency. Serum lead, assayed during a workup for anemia, was noted to be normal. CT of the head and neck area showed a metallic object measuring 19 mm × 7 mm located in the right parapharyngeal space at the level of the second cervical vertebra (Figure 1), along with an old dislocated fracture of the left medial orbital floor (Figure 2).

**Figure 3:**
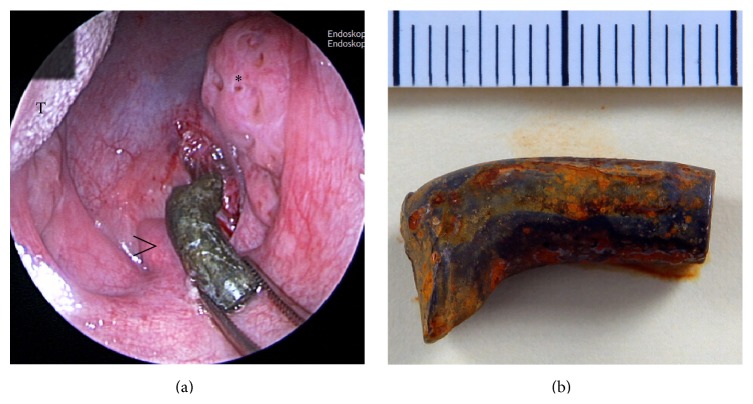
Intraoperative picture showing the extraction of the bullet (a); asterisk: right tonsilla palatina, arrowhead: uvula, and T: tongue. The tip and the jacket of the extracted bullet were torn off (b). The steel core was deformed and the middle portion was bent approximately 45°.

The patient underwent uneventful transoral extraction of the bullet under general anaesthesia (Figure 3(a)). The extracted bullet proved to be a steel core, with a diameter between 6.7 mm and 6.8 mm at the base (Figure 3(b)). The core was deformed and bent approximately 45°. The tip and the jacket were torn off. The entire bullet fragment measured 19 mm in length.

The patient was discharged one day after surgery in good condition. His throat pain resolved completely within a month.

## 3. Discussion

There have been two published cases of a bullet lodged in the parapharyngeal space; however, none of them is describing a migrating bullet [11, 12]. The appearance of the extracted bullet fragment and the fact that a bullet remains within the body raise the question whether banned military ammunition was used. Standard assault rifle ammunition, such as modern NATO (223 Rem 5.56 mm × 45 mm) and Kalashnikov (5.45 mm × 39 mm) ammunition, is characterized by very high muzzle velocity (965 m/s and 900 m/s, resp.) and generally passes through human targets. Deforming ammunition in a military setting constitutes a violation of the Hague Convention of 1899 (Laws of War: Declaration on the Use of Bullets Which Expand or Flatten Easily in the Human Body; July 29, 1899) and in effect constitutes a war crime. Hence, (war) surgeons are often asked whether such ammunition was used or not and profound knowledge in the field of wound ballistics is important.

In our case, however, the extracted object was a steel core (the gilded metal jacket had been stripped off). Based on the diameter, it is most likely a Kalashnikov model M74 (5.45 mm × 39 mm). The bent shape and the stripped-off jacket strongly indicate that the bullet struck a hard structure on its course, thus making it a ricochet. Wounds caused by ricochets show completely different ballistic characteristics than wounds caused by bullets striking a primary target. One of the most notable differences is that the projectile's further flight is unstable. Bullets may lose kinetic energy by ricocheting and by losing mass, which both occurred in this case. This explains why our patient presented with a retained standard military full metal jacketed (FMJ) bullet, as the pierced orbital and pharyngeal walls do not constitute any real resistance to a projectile with a high muzzle energy (>1600 J). However, if the bullet had not lost a lot of its energy or if it had hit the patient directly, it would most likely have penetrated the whole cranium, causing extensive, almost certainly fatal, injuries.

The presented case raises another question, namely, how the bullet may have ended up in the parapharyngeal space after obviously striking the left bulbus oculi. There are two major ballistic aspects to be taken into consideration: immediate bullet course and migration, which have been described for many different kinds of foreign bodies [13–17]. The immediate bullet course can lead to a highly unexpected end position of the projectile [18]. It is a general misconception that a bullet will take a more or less straight course in soft tissue between the entrance and the end position. This is likely in the case of short projectiles such as revolver and pistol bullet. Long FMJ projectiles, however, generally take a bent or warped course, which is especially true in the case of wobbling ricochets. Therefore, the end position of the bullet in the parapharyngeal region very distant to the entrance site may be possible without migration. There was no radiologic evidence for internal ricocheting, for example, after striking a hard bone.

Another possible, albeit straight, bullet course based on CT imaging may start at the left bulbus oculi and end in the right upper parapharyngeal space after passing through the left medial orbital floor, the left nasal cavity, and the pharyngeal lumen. Such a short wound channel may only be explained by a low kinetic energy of the bullet, naturally accompanied only by minor lesions to the surrounding tissue. This is consistent with our case, as, concluded by its appearance, the bullet must have lost a lot of its energy by ricocheting and by losing mass. Moreover, the only obvious internal injury was a dislocated orbital floor fracture. However, as the patient presented 8 months after the accident, minor injuries of the soft tissue may have already healed completely. Thus, a short wound channel ending in the upper pharynx, more cranial than the bullet which was found later, is very plausible from a ballistic point of view. It is therefore highly probable that the deformed, unstably moving low-energy bullet was initially lodged somewhere in the upper pharyngeal structures before migrating caudally.

When evaluating these two hypotheses explaining the position of the bullet, that is, immediate but warped course of the bullet versus rather straight course and subsequent migration of the bullet, the clinical context has to be taken into account. The patient's history is suggestive of bullet migration. He convincingly described a different position of the pain and foreign body sensation over time, which he interpreted as bullet migration. Secondly, no skin or mucosal lesions suggestive for a gunshot wound were found upon HEENT examination. Moreover, the patient suffered from a constant and severe sore throat that may have been partly caused by the gradual movement of the bullet inducing a constant inflammatory reaction. Lastly, a bullet entering through the open mouth seems unlikely given the obvious injury of the left eye, followed by internal injuries (dislocated fracture of the orbital floor) along the route to the pharynx. All in all, there is no clinical evidence suggesting that the bullet took a direct course to its final location.

Our case illustrates that bullets may take an unexpected course once having entered the body, especially if they are ricochets. Bullets are able to change location within the body by migrating, rendering ballistic reconstruction even more difficult.
